# Face expressive lifting (FEL): an original surgical concept combined with bipolar radiofrequency

**DOI:** 10.1007/s00238-013-0908-2

**Published:** 2013-12-06

**Authors:** Marc Divaris, Guillermo Blugerman, Malcolm D. Paul

**Affiliations:** 1 Department of Plastic and Maxillo-Facial Surgery, University of Pitie Salpetiere, Paris, France; 2 Clinica B&S De Excelencia en Cirurgia Plastica, Buenos Aires, Argentina; 3 Department of Surgery, Aesthetic and Plastic Surgery Institute, University of California, Irvine, CA USA

**Keywords:** Face Expressive Lifting (FEL), Bipolar radiofrequency, Skin tightening, Facial surgery, Facial expression

## Abstract

**Background:**

Aging can lead to changes in facial expressions, transforming the positive youth expression of happiness to negative expressions as sadness, tiredness, and disgust. Local skin distension is another consequence of aging, which can be difficult to treat with rejuvenation procedures. The “face expressive lifting” (FEL) is an original concept in facial rejuvenation surgery. On the one hand, FEL integrates established convergent surgical techniques aiming to correct the age-related negative facial expressions. On the other hand, FEL incorporates novel bipolar RF technology aiming to correct local skin distension.

**Methods:**

One hundred twenty-six patients underwent FEL procedure. Facial expression and local skin distension were assessed with 2 years follow-up.

**Results:**

There was a correction of negative facial expression for 96 patients (76 %) and a tightening of local skin distension in 100 % of cases.

**Conclusions:**

FEL is an effective procedure taking into account and able to correct both age-related negative changes in facial expression and local skin distension using radiofrequency.

Level of Evidence: Level IV, therapeutic study.

## Introduction

The recent years have seen a better understanding of the physiopathogenesis and manifestations of facial aging such as sagging due to gravity, fat loss and redistribution, and loss of bone volume [1–5].

However, aging can also lead to changes in facial expressions, transforming the positive youth expressions (happiness) to negative expressions (sadness, tiredness, and disgust) overtime [6, 7]. These projected “negative emotions” can impact other's views of us and social interaction. This might explain that a high number of patients ask not only for cosmetic improvement but also for improvement of unattractive facial expression.

Almost no studies have clearly analyzed and shown how to correct the age-related changes on facial expression.

The FEL concept was developed upon the convergence of effects of rejuvenation surgical procedures on different levels of the face with priority given to the re-establishment of “vitality” in both the eyes and the smile, which are the key of emotions expressions.

The present study aimed to address the question of wether the FEL could correct negative facial expressions of the aging face.

Secondly, the skin of the face loses its tonicity over time. Although a lift can eliminate remaining folds after musculocutaneous tightening, simple redraping is not always sufficient in cases of local distension.

The application of bipolar radiofrequency (RF), using FaceTite™ (Invasix Ltd., Yokneam, Israel) makes it possible to obtain stable quantitative and qualitative effects on the skin over time through the regeneration of the deep dermis and superficial subdermal septo-fascial fat [12].

The second question addressed was whether we could correct local skin distension on zones of weak facial skin (malar crescent, labio-jugal, and submental) using radiofrequency.

## Materials and methods

The FEL technique has been applied to 155 patients (128 women and 27 men), ranging from 27 to 82 years old (average age, 54), between March 2010 and December 2010.

FEL aims to correct an almost constant set of aging signs that are systematically tackled.

Indications for FEL treatments were the following:

- A “sad” or “tired” expression of the eyes and smile (Clinical case 1a). Most of our patients complained of these negative expression (91 %).

The eyes:

- Ptosis of the lateral eyebrows and upper eyelids
- Tear through depression under the eyes.

**Fig. 1:**
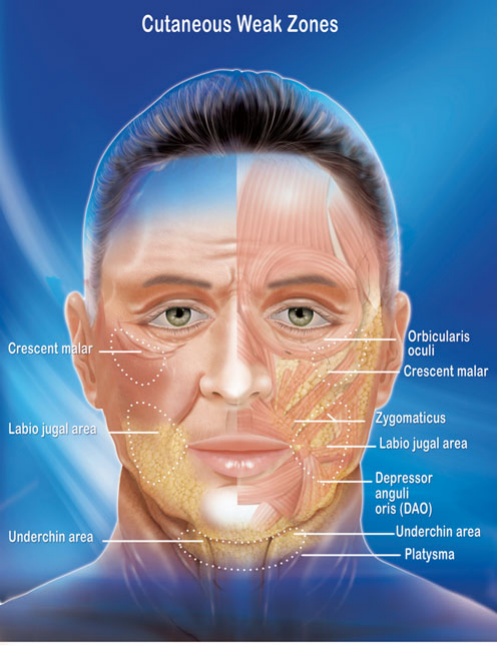
Three weak zones of the face: malar crescent, labio-jugal and submental. These zones maintain a cutaneous memory despite the redistribution effect of the lift. Only a local deep contracting action will correct or prevent this local distension of the skin

The smile:

- Lengthening and thinning of the upper lip
- Downward turn of the upper lip with disgust expression.

- Local skin distension of the three weak facial zones: the malar crescent, the labio-jugal zone, and the submental (Fig. 1). This local distension of the aging skin result from muscular contentions, fat superficialization and gravity [2].

Only the patients who had pre- and postoperative photographs and examinations at 1 month, 3 months, 1 year, and 2 years were included in this study.

Patients utilized the linear analogue scale to evaluate facial expression improvement whereby poor and excellent results scored respectively 1–5.

Analysis and comparison of pre- and postoperative photographs and examinations were made by the surgeon assessing:

- Neutral negative facial expression correction (yes/no)
- Correction or not of local skin distension at the site of RF application (the preventive RF application done for malar crescent zone where there was no distension was not included in the study).

All the techniques used integrate known surgical methods, albeit modified, with RF technology. The novel technology that is combined with these techniques is the facial bipolar RF by FaceTite™ applied on the three weak facial zones. This device's precision makes it possible to custom treat the following three weak facial zones (Fig. 1) to both qualitatively and quantitatively eradicate damaged skin memory, leading to skin tightening.

The multispot heating by the RF on the deep surface of the dermis and subdermis allows coagulation and long-term stable tightening.

### The device

Patients were treated in the facial areas using the FaceTite™ device employing RF-assisted liposuction technology. The FaceTite hand piece is affixed with two electrodes, one external and one internal, to provide dermal and subdermal heating resulting in skin contraction. RF energy is generated at a frequency of 1 MHz within a power range of 10–25 W. The RF current travels between the electrodes, coagulating adipose tissue, and providing controlled heating of the dermal and subcutaneous collagenous tissue. The internal electrode, acting at the tip as an energy source and having a 1.5-mm diameter, 10-cm long cannula, is inserted through a stab wound incision. The 1-cm disc-like external electrode is applied to the skin surface directly above the RF-emitting tip of the internal electrode, and RF is alternating between the two electrodes.

During the procedure, the used treatment parameters were treatment depth (∼3 mm—superficial subdermal plane), RF power (10–15 W) and skin surface temperature (38–40 °C), which are controlled by the user.

### Surgical technique

The intervention is carried out under general anesthesia followed by tumescent infiltration.

**Fig. 2:**
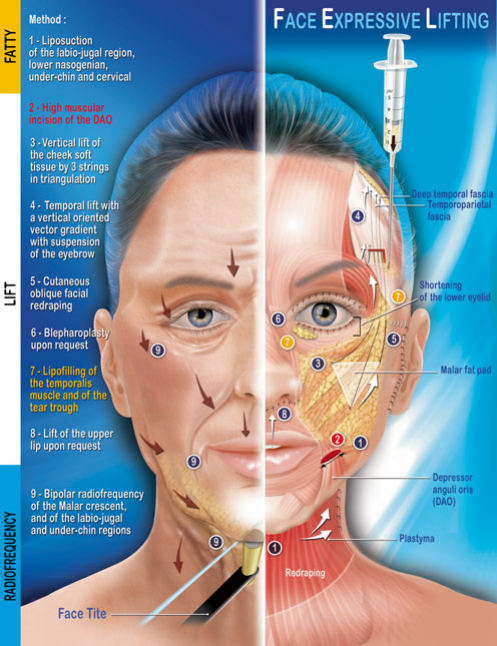
Face Expressive Lifting (FEL): complete procedure in nine multifactorial stages indicating the facial parts and the various tissues

The stages of the FEL procedure (Fig. 2) are as follows:

1. Liposuction

Procedure was carried out using a Byron-type cannula, mounted on a syringe inserted via bilateral temporal incision at the root of the hair and under the chin. Lifting the cheeks and removing the fat from labio-jugal, submental, and neck zones create a youthful fullness by reducing the width of the lower level of the face. On the contrary, the upper zones of the cheeks and the supra nasolabial fold areas were left untouched, since fat removal from these areas would diminish the youthful fullness.

2. Severing of the depressor anguli oris (DAO) muscle

This was carried out following a low vestibular intra-jugal incision. The detected DAO muscle was severed and coagulated transversally, which immediately relieved the downwards pull.

3. Cheek lift

**Fig. 3:**
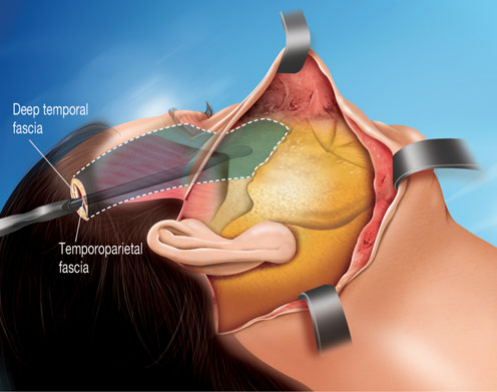
Lateral view 1: the tissue of the malar unit is customized by deep subperiosteum and superficial subcutaneous detachment. The end of the eyebrow region is detached over periosteum to facilitate its vertical mobilization

A short 2-cm horizontal temporal scalp incision was made at the level of the upper part of the temporal fossa followed by dissection inferior to the temporal fascia and superior to the temporal aponeurosis, releasing the lateral orbital rim, and identifying the medium deep temporal vein (Fig. 3). The dissection was then extended downwards subperiostally at the elevator, affecting the entire zygomatic area and the inferior orbital rim, to easily mobilize the entire fat, muscle, and skin entity.

**Fig. 4:**
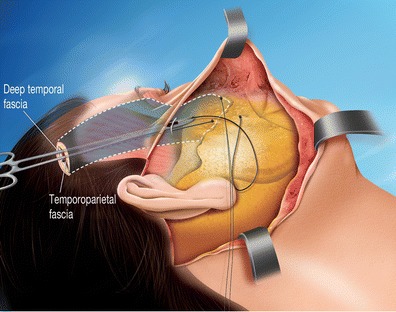
Lateral view 2: the tripod of soft malar tissue is suspended via three angles using a woven string rejoining the upper deep detachment plane through a decongestive cutaneous incision made in the fascia without damaging the facial nerve

**Fig. 5:**
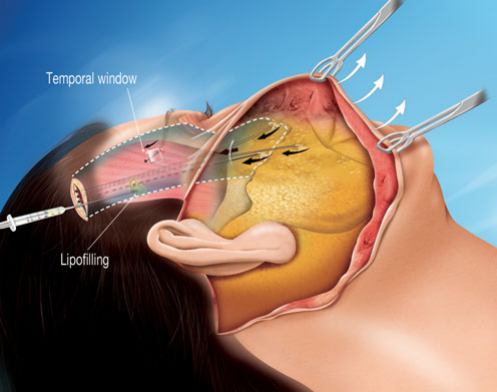
Lateral view 3: the vertical mobilization of the core of the cheek is very easy and its positioning is adjusted according to the degree of tightening of the threads on the temporal aponeurosis. The lower eyelid can also be shortened upon request. A temporal window with an inferior flap (junction at bottom) is created for the positioning of the eyebrows. This is lifted in a customized manner by a running thread that catches the deep dermis of the eyebrow and sutures it to the upper part of the window. The scarring through a fake muscle plane creates scar tissue adhesions, stabilizing the result. Before closing the temporal window, a centrifuged lipofilling is injected beneath the temporal aponeurosis to plump up the temporal fossa

Using the preauricular incision, the subcutaneous dissection reached the malar area, isolating the entire superficial and deep fatty compartment of the cheek that is separated by the malar septum [4] and then suspended with triangular anchoring directly to the temporal aponeurosis by three woven resorbable strings (Vicryl 2/0). Two superior points at the upper extremities of the isolated malar entity and one inferior point at the lowest median point of this entity (Fig. 4). These suspensions support the essential points (Fig. 4) and pass through the temporal fascia, then join the dissected temporal plane and attached to the temporal aponeurosis (Fig. 5). The superior malar double suspension ascends the cheek, while the lower malar suspension relieves the superior labio-jugal region.

The cheek lift makes it possible to ascend the tissue mass of the cheek by several millimeters to a few centimeters, adjusted as needed to reposition the cheek in a vertical axis. The oblique facial cutaneous redraping was subsequently carried out through preauricular incision ideally completes this gesture as it liberates any possible indentations caused by the previous actions and three dimensionally sets the cheek into its new position.

4. Brow lift

Suturing the lateral edge of the eyebrow at the level of the 1-cm^2^ temporal window flap with an inferior hinge was completed, using a 3/0 silk thread. In the lateral edge of the eyebrow, the needle passes through the dermis and the skin and goes back by the same hole in order to make a loop providing solid anchorage. This positioning is durable and the suspension is held in place, by the involvement of the deep dermis and the resulting scarring fibrosis.

5. Conventional cervicofacial lift

**Fig. 6:**
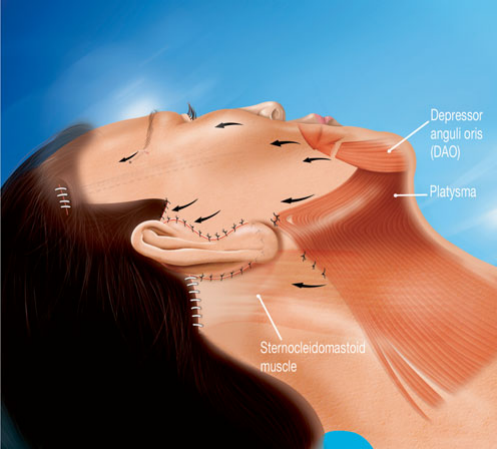
Lateral view 4: all volume correction axes and muscle tractions are *vertical*. It is more the vertical axis rather than the traction force that guarantees a natural look. It is important to recognize the combination of effects: the inferior thread of the malar tripod through its elevation of the modiolus makes the spacing between the two stumps of the DAO long lasting, even augmenting their spacing

A preauricular incision prolonged by a horizontal precapillary incision (Mac INDOE), associated or not with a retroauricular incision was made. SMAS and platysma plication was then performed (Fig. 6).

6. Blepharoplasty

Lower blepharoplasty was carried out if needed. Often, lifting the cheek leads to a compression of the skin at the lower eyelid and a blepharoplasty enables the excess skin removal. The noble orbital muscle structure left untouched and only fibers are separated according to the concentric axis in order to access prominent fatty pockets. An upper blepharoplasty often completes the procedure.

7. Temporal and tear through fat transfer

Intramuscular subaponeurotic lipofilling, using autografted stem cells may correct the aging temporal depressed area. In case of a tear through squeletization, a light lipofilling under the orbicular muscle along the anterior lacrimal crest help fill in this depression. We never perform hypercorrection.

8. Upper-lip lift

Upper-lip lift was performed as well in order to revitalize the smile. This was achieved after making a skin excision at the base of the nose. The excision may be shorter or longer, depending on the shape of the nose.

9. RF treatment of the three weak zones

**Fig. 7:**
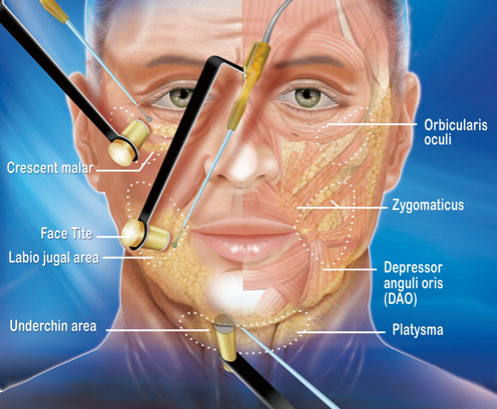
FaceTite: entry point for the hand piece. FaceTite treats the three weak facial zones

These zones were treated by 5–10 targeted shots of FaceTite at a power of 10–12 W in pulsed mode, according to the severity of the local distension( Fig. 7).

## Results

Out of 155 cases that underwent FEL, only 126 of these patients had complete data and have been included in the study.

The combination of FEL surgical procedures performed for the 126 patients is summarized in Table 1.

**Table 1:** Combination of FEL surgical procedures

Number of patients	Cheek lift	Brow lift	SMAS-platysma plication	Liposuction	Tear through and/or Temporal lipofilling	Lower blepharoplasty	DAO procedure	Upper-lip lift	Upper blepharoplasty
2	+	+	+	+	+				+
5	+	+	+	+	+				
6	+	+	+	+	+	+			+
3	+	+	+	+	+	+			
11	+	+	+	+	+	+	+		+
1	+	+	+	+	+	+	+		
36	+	+	+	+	+		+	+	+
8	+	+	+	+	+	+		+	+
28	+	+	+	+	+	+	+	+	+
5	+	+	+	+	+			+	+
7	+	+	+	+	+		+		
14	+	+	+	+	+		+		+

### Facial expression

Patients evaluation of facial expression was excellent (mean score 4.59 out of 5.00).

Analysis photographs and examinations showed a noticeable correction of negative facial expression in 96 patients (76 %), which was stable over time.

All the three weak zones of the face treated therapeutically by FaceTite (322 zones) revealed an impressive tightening in 100 % with no complications. There was also an improvement in skin quality:

- Malar crescent zone: This area was retracted in 100 % of patients: 28 % systematically for prevention, 72 % therapeutically (182 zones). When done therapeutically, the small malar pockets simply disappeared, thus giving the expected positive result and the bigger malar pockets were reduced. Edema has disappeared in this area after 3 months (Clinical case 3).
- Labio-jugal zone: FaceTite RF treatment was done in 30 % (76 zones) of patients, including cases where preoperatory distension increased after liposuction of this area. The triangle effect of the cheek lift was further enhanced by this combination work. Retraction was noticeable and stable during follow-up.
- Submental zone: RF treatment was performed in 51 % (64 zones) of patients when local distension was present and following liposuction. Retraction was stable and no complication occurred.

### Complications

- Hematoma evacuation under local anesthesia was required in two patients with no effect on outcome.
- Three patients had transitory facial nerve dysfunction.
- Three patients required intralesional steroid therapy for scar hypertrophy.
- We have never encountered any problems concerning vascularization.

## Discussion

FEL (Fig. 2) is based on emphasizing the multiple and complementary nature of gestures aimed at enhancing the vitality of the smile and eyes and repositioning the face in its vertical dimension.

FEL integrates various well-known surgical techniques that have proven to be efficient. Some techniques have been modified in order to gain durability and efficiency as listed below.

### Brow lift

The positioning the external edge of the eyebrow, as desired, directly upon the temporal muscle plane (obtained by creating a 1-cm^2^ fascia temporal window), using a suspended thread, is stable over time because of the resulting scar adhesions.

### Cheek lift

This modified cheek lift (Fig. 5) specifically isolates the three layers of the malar entity (superficial fat, aponeurosis, and deep fat) from their superficial attachments (skin) and deep connections (bone).

This double detachment from the bone and skin allows for the combination of a vertical suspension that lifts the cheek and the oblique facial redraping that erases indentations,

The cheek released can be precisely put in an adjustable position taking into consideration the three-dimensional appearance of the zygomatic bone, which is a bony convex area that varies from one side of the face to the other.

Positioning seems easier than when using RARE [9] or Face Recurve-type techniques [2].

According to the strength of the tension of the suspended threads, the height of the lower eyelid can be precisely shortened, with excess skin resected as desired.

### FEL and negative facial expressions

Ekman described the universal facial expression of sadness as a lowering of mouth corners and a raise of inner eyebrows [7]. These features are also found in the aging face.

The two main sources of facial expression are the look of the eyes and the smile that FEL aimed to improve.

Although facial expression evaluation by the surgeon and the patients was subjective, FEL has obviously improved facial expression (Clinical cases 1, 2, and 3) in most patients (76 %).

**Clinical case 1:**
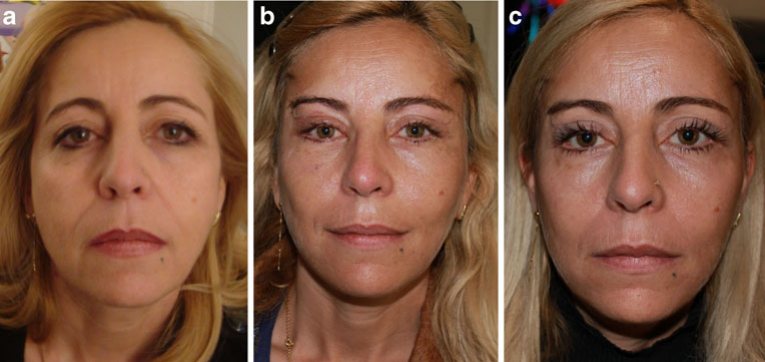
Preoperative 55-year-old woman, presenting sagging in the main axes of the face (eyebrow, cheek, and corner of the mouth) resulting in a sad and negative facial expression (**a**). Seven days follow-up, presenting overcorrection of the main lines in a static position with a natural appearance (**b**). One year follow-up, presenting well-balanced face in a static position with a stabilization of the main facial lines (**c**) and correction of the negative facial expression

**Clinical case 2:**
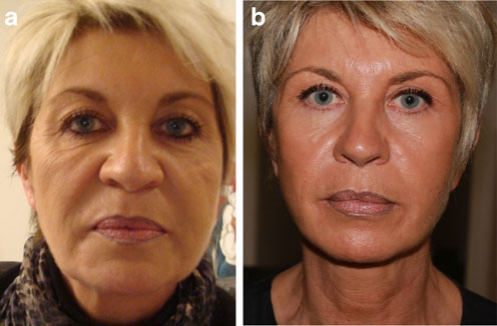
Preoperative 56-year-old woman, presenting all the signs of aging with sad expression of the eyes and disgust expression of the mouth (**a**). Ten months postoperative photo (**b**), following all the nine stages of the FEL, showing facial triangle with correction of negative facial expressions and the three weak zones of the face firmed by RF

**Clinical case 3:**
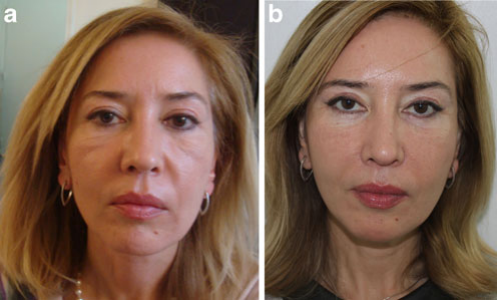
Preoperative 51-year-old woman, presenting a bilateral ptosis of the cheeks with the presence of malar pockets and a heavy labio-jugal region (**a**). One year follow-up, demonstrating stable results over time with a restored triangle, disappearance of the malar pockets, and a decrease in the transversal dimension of the lower face (**b**)

### The look of the eyes

Knoll et al. [8] found that tiredness expression was related to low upper eyelid and depressed lateral eyebrow. This suggests that blepharoplasty and brow lift were the key procedures to turn such an expression into a youthful open look of the eyes.

The restoration of the plumpness of the temporal fossa eradicates sunken areas of the face by giving them fullness and thus might improve that tiredness expression.

In addition, happiness was perceived by elevation of lower eyelid [8]. This suggests that cheek lift by shortening the lower eyelid and restoring face triangularity might create a happier expression.

Support of the lower orbital rim is reconstituted by the lift of the fat of the cheek; however, a slight depression of the under-eyes may persist and is corrected using a light filling performed under the orbicular muscle. This might contribute to a less tired expression.

### The smile expression

The smile expression constantly degrades from about age 50 with downturn and lengthening of the aging upper lip. It is important to combine and choose the various synergetic procedures according to diagnosis, which can include a subnasal lip lift, the upper severing of the DAO, and the tightening of the labio-jugal skin. These different procedures can be employed in association or independently to revitalize the buccal orifice.

In the upper lip lift, its therapeutic diagnosis is the nonvisibility of the upper teeth upon passive opening of the mouth. Performed with a short or long incision along the contours of the base of the nose, the result of this bull-horn subnasal lip lift is very revitalizing and restores equilibrium between the white lip and red lip.

The downward turn of the oral commissure often is not affected by the SMAS techniques. The DAO procedure does an excellent job in correcting the disgust expression and freeing up the smile. The upper section of the muscle was potentiated by the vertical tension of the malar tripod, which accentuated the spacing of the muscle stumps and made the result long lasting (Clinical case 2).

We believe DAO procedure is commonly required after 50 years of age because of the natural downward collapse of the superior lip because of the activity of the tonic DAO's muscular fiber.

### Local skin distension

In our study, it was possible to correct selectively, in a customized manner, zones of local facial skin distension (malar crescent, labio-jugal and submental) and to obtain stable quantitative and qualitative effects on the skin over time.

Reinforcing the qualities of the deep dermis [10–12] acts as a curative and preventive measure.

Increased tightening by heat and coagulation of the collagen matrix occurs in the deep dermis and the upper septa with regeneration over a period of 8–12 weeks, improving the quality of tissues with stable tightening and even slow down the sagging process over the forthcoming years.

In accordance with other clinical data on the subject [10–12], the firming effect of RF by deep coagulation made quantitative contraction possible by 10–15 % between two points (scar and benign tumor) depending on skin type. These publications demonstrated bipolar RF's ability to contract the skin from 15–30 %, depending on the zone treated, RF strength, and delivered energy.

### The malar crescent

This anatomic entity presents a very thin skin that is prone to distension. It is sometimes visible prior to surgery but can sometimes appear at postoperative stages following blepharoplasty, along with edematous and ecchymotic phenomena. FaceTite treatments were routinely carried out with variable parameters, taking into consideration the thickness of the skin. The disappearance or softening of these malar pockets makes it possible to improve the look of the eyes. The result is effective, quickly obtained, and prevents the appearance of these postlift edematous and ecchymotic phenomena in the long-term postsurgery.

### The labio-jugal zone

This region is represented by local hypotonic skin and abundant superficial fat that is carefully removed by liposuction. The RF technology is very useful at this point.

Firming the skin of the labio-jugal area perfected the desired triangular shaping of the face.

### The neck

The redistribution of the neck tissue by various lifting does not always restore the skin tone provided by the deep dermis gravity [13].

Complementary with the classical neck lift, FaceTite makes it possible to obtain a tightening of the neck region, a thickening of the deep dermis, and a deep attachment to the deep scar tissue caused by controlled burning.

RF is a novel tool in neck rejuvenation for moderate skin neck ptosis and may help avoiding heavier procedures.

## Conclusion

The main objectives of this original FEL concept included the tightening of local skin distension to the three key points of the face and correction of age-related negative face expressions. This was achieved through converging procedures with multifactorial work on the tissues.

For the first time, a procedure takes the age-related negative facial expressions into account with priority and is able to correct them.
